# Adaptations to the first wave of the COVID-19 pandemic by private sector tuberculosis care providers in India

**DOI:** 10.1016/j.jctube.2022.100327

**Published:** 2022-07-19

**Authors:** Shamim Mannan, Charity Oga-Omenka, Akhil Soman ThekkePurakkal, Lavanya Huria, Aakshi Kalra, Ravdeep Gandhi, Tunisha Kapoor, Nathali Gunawardena, Shekhar Raj, Manjot Kaur, Angelina Sassi, Tripti Pande, Vijayan Shibu, Sanjay Sarin, Sarabjit Singh Chadha, Petra Heitkamp, Jishnu Das, Raghuram Rao, Madhukar Pai

**Affiliations:** a Clinton Health Access Initiative (CHAI), India; b McGill International TB Centre, Montreal, Canada; c School of Public Health Sciences, University of Waterloo, Canada; d Foundation for Innovative New Diagnostics (FIND), India; e Department of Epidemiology, Biostatistics and Occupational Health, McGill University, Canada; f PATH, India; g Centre for Health Research and Innovation (CHRI), India; h TB PPM Learning Network, Research Institute of the McGill University Health Centre, Canada; i McCourt School of Public Policy, Georgetown University, Washington, DC, USA; j Central TB Division, Ministry of Health & Family Welfare, India

**Keywords:** Private sector providers, TB, Diagnosis and treatment, COVID, India, Adaptations, Healthcare delivery

## Abstract

**Background:**

India’s dominant private healthcare sector is the destination for 60–85% of initial tuberculosis care-seeking. The COVID-19 pandemic in India drastically affected TB case notifications in the first half of 2020. In this survey, we assessed the impact of the first wave of COVID-19 in India on private providers, and changes they adopted in their practice due to the pandemic.

**Methods:**

The Joint Effort for Elimination of TB (JEET) is a nationwide Global Fund project implemented across 406 districts in 23 states to extend quality TB services to patients seeking care in private sector. We conducted a rapid survey of 11% (2,750) of active providers engaged under JEET’s intense Patient Provider Support Agency (PPSA) model across 15 Indian states in Q1 (February–March) of 2021. Providers were contacted in person or telephonically, and consenting participants were interviewed using a web-based survey tool. Responses from participants were elicited on their practice before COVID-19, during the 2020 lockdowns (March–April 2020) and currently (Q1 2021). Data were adjusted for survey design and non-response, and results were summarised using descriptive statistics and logistic regression.

**Results:**

Of the 2,750 providers sampled, 2,011 consented and were surveyed (73 % response). Nearly 50 % were between 30 and 45 years of age, and 51 % were from Uttar Pradesh, Maharashtra and Gujarat. Seventy percent of providers reported reduced daily out-patient numbers in Q1 2021 compared to pre-COVID times. During the lockdown, 898 (40 %) of providers said their facilities were closed, while 323 (11 %) offered limited services including teleconsultation. In Q1 2021, 88 % of provider facilities were fully open, with 10 % providing adjusted services, and 4 % using teleconsultation. Only 2 % remained completely closed. Majority of the providers (92 %) reported not experiencing any delays in TB testing in Q1 2021 compared to pre-COVID times. Only 6 % reported raising costs at their clinic, mostly to cover personal protective equipment (PPE) and other infection control measures, although 60–90 % implemented various infection control measures. Thirty-three percent of TB providers were ordering COVID-19 testing, in addition to TB testing.

To adapt, 82% of survey providers implemented social distancing and increased timing between appointments and 83% started conducting temperature checks, with variation by state and provider type, while 89% adopted additional sanitation measures in their facilities. Furthermore, 62% of providers started using PPE, and 13% made physical changes (air filters, isolation of patient areas) to their clinic to prevent infection. Seventy percent of providers stated that infection control measures could decrease TB transmission.

**Conclusion:**

Although COVID-19 restrictions resulted in significant declines in patient turn-out at private facilities, our analysis showed that most providers were open and costs for TB care remained mostly the same in Q1 2021. As result of the COVID-19 pandemic, several positive strategies have been adapted by the private sector TB care providers. Since the subsequent COVID-19 waves were more severe or widespread, additional work is needed to assess the impact of the pandemic on the private health sector.

## 1 Background

India has the world’s highest burden of tuberculosis (TB), and has been significantly affected by COVID-19 (Fig. 2a) [1], [2], [3]. India’s dominant private healthcare sector accounts for approximately 74 % of initial TB care-seeking and 54 % of all TB drug distribution, and about two-thirds of patients continue their care with private sector [4], [5], [6]. Yet, the private sector accounted for less than a third of the 2019 TB notifications [4].

**Fig. 1:**
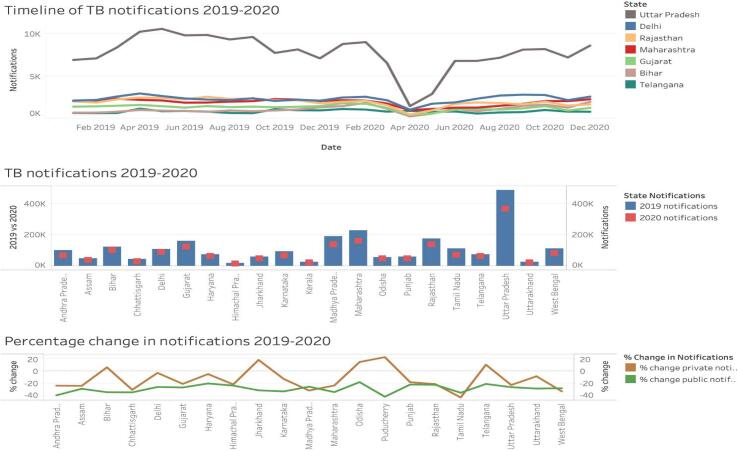
TB notifications timelines and trends in selected States, 2019–2020.

As a direct result of the lockdowns and diversion of resources due to the first wave of COVID-19 pandemic, India’s TB case notifications dropped by 25 % in 2020 compared to 2019 [1], [7], [8]. The largest decline was documented during the months when India imposed strict lockdown (March-April) in 2020. The private sector experienced a significant drop of 44 % in TB case notifications during these months compared to January-February 2020 (Fig. 1) [8].

Although lockdown protocols and restrictions were implemented to limit the spread of COVID-19 throughout the country, they inadvertently caused disruptions in the TB care-seeking and service delivery [3]. The pandemic affected TB providers on several fronts, as 78 % of Global Fund supported providers reported significant disruptions [9], and 44 countries around the world reported partial disruption for TB case detection and treatment in a key informant survey conducted by the WHO [2]. However, not enough is known about the specific ways in which providers have adapted their service delivery following COVID-19, particularly in the private sector.

Understanding any COVID-19 related changes in private TB service provision is a necessary step to developing targeted mitigation strategies. The Joint Effort for Elimination of Tuberculosis (JEET) project is the largest private health sector engagement initiative for TB in India [10]. JEET is implemented by three Global Fund Principal Recipients, Centre for Health Research and Innovation (CHRI), Clinton Health Access Initiative (CHAI), and Foundation for Innovative New Diagnostics (FIND), together with 8 Sub-Recipients, across 200,000 private providers in 406 districts in 23 states since 2018. Operationalised through a resource intensive Private Provider Support Agency (PPSA) model and resource limited PPSA lite model, the project’s objective is to establish sustainable connections between private health facilities, chemists, laboratories and the national public health program. The JEET initiative focuses on extending quality TB services to patients seeking care in the private sector, building upon successes and learnings from previous projects [10]. Additional details of this initiative and the PPSA implementation model has been published elsewhere  [11]. Utilising the network of private sector providers established by JEET, the objective of this cross-sectional survey was to assess the impact of COVID-19 on the private sector focusing on TB services and the consequent adaptations made by providers in their care practice. The survey was conducted by JEET partners as part of their routine service delivery work, to assess if COVID disruptions were impacting their ongoing program.

## 2 Methods

### 2.1 Survey design, study sites and participants

The rapid survey included providers across 15 states in India (Fig. 2) and was conducted in Q1 (February-March) of 2021 (Fig. 2a). A stratified random sampling technique was used to select the participants. The sampling frame (N = 25,279) constituted all providers (private health facilities, chemists, and laboratories) who were sensitized and engaged by the JEET partners and who notified TB cases in 2020. From a total of 23 states covered by JEET, 8 were excluded because they did not have the more intensive (PPSA) activities in 2020. Within the strata represented by each of the three JEET partners (CHAI: N = 4,690, PATH: N = 6,705, FIND: N = 13,884), we randomly sampled N = 2,750 providers (CHAI: N = 1051, PATH: N = 999, FIND: N = 700) with the aim of covering at least 10 % of the sampling frame and to account for non-response (based on pilot results) while considering variability in provider type and partner resources. Sampling was done using statistical software (R, version 4.0.2). We also ensured that at least 5 providers were surveyed from each district (N = 75) within each of the 15 states.

**Fig. 2a:**
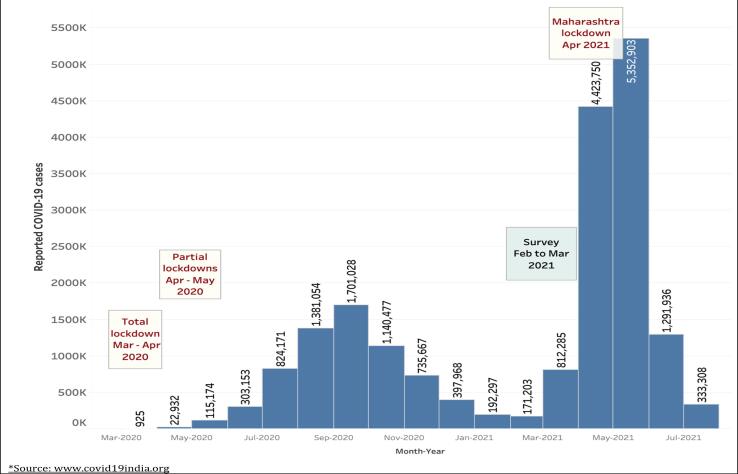
*Epi*-curve of confirmed reported COVID-19 cases in India showing survey start and end dates*.

### 2.2 Data collection

The survey questionnaire was developed and revised with active participation from all partners and pilot-tested in January 2021. Questions covered aspects of provider practice before COVID-19, during the first lockdown period in India (25 March 2020 – 31 May 2020), and current practice (Q1 2021), covering several domains including outpatient visits, TB diagnosis, treatment and care costs, patient care and opportunities to improve TB care.

To collect data, the structured questionnaire was administered to the providers by JEET field officers who were systematically trained on administering the survey by each partner. These field officers contacted the providers in-person or by telephone as part of their routine service calls. A maximum of three attempts were made to contact each provider, after which non-contactable providers were classified as non-responders. Consenting participants were interviewed using the survey tool deployed through the Kobo Toolbox data collection platform [12].

### 2.3 Statistical analysis and survey weighting

Prior to analysis, we weighted the data to adjust for survey design (stratified simple random sampling within the strata of partners) and non-response. To account for survey design, we calculated sample weights (w1) as the inverse probability of selecting providers with each JEET partner [13]. This accounted for provider sampling variations and the disproportionate sampling stratification among the partners. The sum of the sample weights equaled the total sampling frame units (N = 25,279), and sum of normalized weights equaled the sample size of responders (N = 2,011). We also weighted the sample for non-response (27 %) to ensure that the responders resembled the original sample using known characteristics [13], [14]. The non-response weights (w2) were calculated from the predicted probabilities (P) of being a respondent in the survey. The predicted probabilities were estimated by fitting a logistic regression model for response using auxiliary variables (partner organizations, state/province of survey, type of providers) and paradata (number of attempts to contact a provider) that were available commonly across both responders and non-responders. The unweighted mean of the predicted probabilities equaled the unweighted response rate in the survey (73 %) [15]. The weight, w1, was calculated as 1/P for responders [15]. The final non-response adjusted weight (W) that was used in the descriptive and predictive models for the analyses was calculated as the product of the sample weight and the non-response weight (W = w1*w2). Sampling and response weights per partner are shown in Appendix I. Additionally, as more than 5 % of the sampling frame was sampled in the survey, a finite population correction factor was also included in the models [13].

Data presented in this manuscript have been described using frequencies and weighted proportions. Unconditional logistic regression models were fitted to estimate the weighted odds ratios (OR) and 95 % confidence intervals (CI) to identify predictors of providers being open during the lockdown and in early 2021. These predictors included age of the provider (as age has been associated with COVID-19 morbidity and mortality), provider type, and state. We used<30 years (smallest category in age), health facility (largest category in type of provider) and Uttar Pradesh (largest category in state of the provider) as reference groups. The models were mutually adjusted for these three variables of interest which also acted as confounders. Quantitative data were analyzed using Stata, version 13SE (StataCorp. 2013, College Station, TX), Microsoft Excel 365 and Tableau 2020.4.2.

## 3 Results

### 3.1 Provider characteristics

A total of 2,011 responses (73 % response rate) and 739 nonresponses were tracked and analyzed (Fig. 2b). The number of respondents per state is shown in Fig. 2c.

**Fig. 2b:**
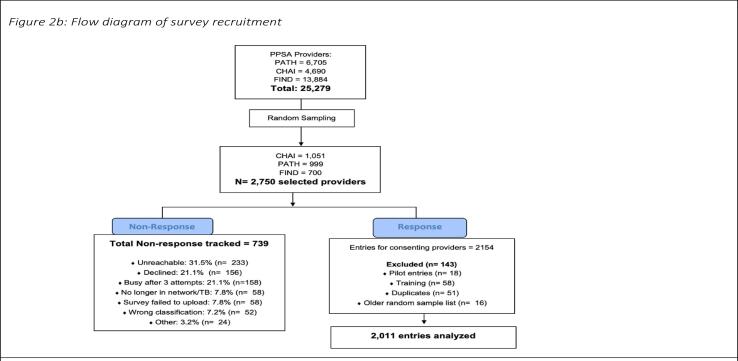
Flow diagram of survey recruitment.

**Fig. 2c:**
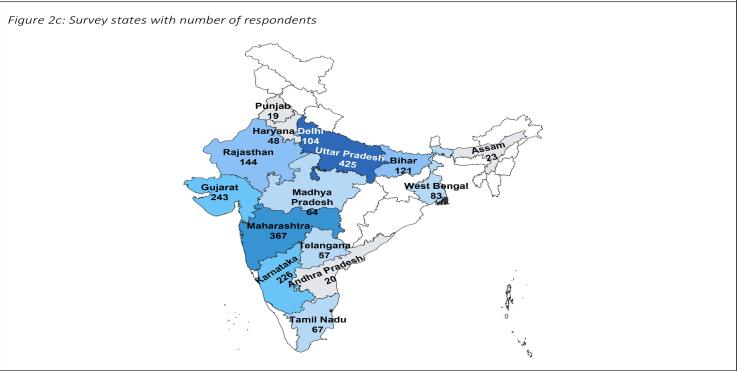
Survey states with number of respondents.

The characteristics of sampled providers disaggregated by partner, type of facility, number of survey contact attempts, and state are presented in Table 1.

**Table 1:** Description of JEET survey providers.

	**Surveyed**		**Not surveyed**		**Total**	
	**N = 2011**	%*	**N = 739**	%*	**N = 2,750**	%*
**Age**						
<30 years	40	2%	NA	NA	40	1%
30-45 years	969	48%	NA	NA	969	35%
46-60 years	773	38%	NA	NA	773	28%
>60 years	229	11%	NA	NA	229	8%

**Partner**						
PATH	815	41%	184	25%	999	36%
CHAI	791	39%	260	35%	1051	38%
FIND	405	20%	295	40%	700	25%

**Type of facility**						
Health Facilities	1885	94%	611	83%	2496	91%
Chemists	91	5%	99	13%	190	7%
Laboratories	35	2%	29	4%	64	2%

**Number of attempts**						
1	1829	91%	628	85%	2457	89%
2	141	7%	64	9%	205	7%
3	41	2%	47	6%	88	3%

**Partner and state**						
**PATH**						
Uttar Pradesh	425	21%	98	13%	523	19%
Maharashtra	367	18%	86	12%	453	16%
Assam	23	1%	0	0%	23	1%

**CHAI**						
Gujarat	243	12%	48	6%	291	11%
Rajasthan	144	7%	30	4%	174	6%
Delhi	104	5%	44	6%	148	5%
Bihar	121	6%	10	1%	131	5%
Madhya Pradesh	64	3%	63	9%	127	5%
Tamil Nadu	67	3%	51	7%	118	4%
Haryana	48	2%	14	2%	62	2%

**FIND**						
Karnataka	226	11%	130	18%	356	13%
West Bengal	83	4%	84	11%	167	6%
Telangana	57	3%	58	8%	115	4%
Andhra Pradesh	20	1%	17	2%	37	1%
Punjab	19	1%	6	1%	25	1%

*All proportions presented here are unweighted.

In our sampling frame, there were higher proportions of providers from Uttar Pradesh and Maharashtra states; and from health facilities compared to laboratories or chemists selected proportional to their distribution in the sampling frame. Nearly half (48 %) of consenting providers were between the ages of 30 to 45 years. Of the 739 non-responses, 233 (31.5 % of non-responses, 8 % of total sample) were unreachable phone numbers, closed facilities or untraceable addresses. Of those providers who were reached, 158 (21.1 % of non-responses, 6 % of total) declined, 156 (21.1 % of non-responses, 6 % of total) were still busy after 3 attempts to schedule a survey appointment, 58 (8 % of non-responses, 2 % of total) surveys did not upload due to network errors, 58 (8 % of non-responses, 2 % of total) were providers who were no longer in the JEET network or seeing TB patients. Other reasons including field errors and wrong provider classifications accounted for the remaining 76 (10 % of non-responses, 3 % of total sample) of attempts.

### 3.2 Status of providers during and after the widespread lockdowns

Fifty-seven percent of surveyed providers were fully open during the 2020 lockdown period and 88 % were fully open in Q1 2021 (Table 2).

**Table 2:** Unweighted and Weighted Responses to Survey Questions.

**Questions (N = total number of respondents)**	**Responses**	N=2011*	**Unweighted proportions**	**Weighted proportions**
#What was the status of your practiceduring he COVID-19 lockdown? (N = 2006, Missing data = 5)	Open fully for old and new patients in-person	1,054	53%	57%
	Completely closed	898	45%	40%
	Teleconsultations only for old and new patients	323	16%	11%
	Services for existing patients only (including teleconsultations)	117	6%	4%
#What is the status of your practice currently (Q1 2021)? (N = 2005, Missing data = 6)	Fully open/In person visits	1,691	84%	88%
	Adjusted opening hours	282	14%	10%
	Tele-consultation	122	6%	4%
	Completely closed	26	1%	2%
Is your facility outpatient visits higher/lower than before COVID-19? (N = 2011)	Lower	1,350	67%	70%
	Same as before	440	22%	22%
	Higher	201	10%	6%
	Do not know	20	1%	2%
#What services were you offering before COVID-19? (N = 2006, Missing data = 5)	TB testing (e.g. x-ray, histopathology, sputum testing or collection) **	1,776	89%	72%
	Providing TB medication (pharmacy, JEET FDCs or public sector)	1,612	80%	66%
	E-health (tele-consultation, e-pharmacy)	176	9%	9%
	Home consultation	87	4%	7%
	Other	144	7%	12%
#What services are you offering currently (Q1 2021)? (N = 1997, Missing data = 14)	Providing TB medication in-person	1,141	57%	40%
	COVID-19 and TB test together **	960	48%	33%
	TB test alone **	701	35%	27%
	No services	145	7%	20%
	E-health (tele-consultation, e-pharmacy) **	187	9%	8%
	Home consultation	52	3%	3%
	COVID-19 test alone	6	0.3%	0.1%
#** What are the top three advantages to telemedicine or Video DOTS? (N = 174)	Easier to schedule appointments	142	82%	78%
	Reduced transport time & costs	144	83%	73%
	Ability to connect with populations in remote areas/marginalized	117	67%	55%
	Immediate data feedback and ability to report to NTP faster	41	24%	24%
#**What are the top three challenges to telemedicine or Video DOTS? (N = 174)	Internet connectivity	129	74%	72%
	Absence of patient examination by provider	117	67%	58%
	No mobile phone available (provider or patient)	67	39%	30%
	Challenges with digital payments	62	36%	23%
	Patients miss the personal attention and it's difficult to share emotions via telemedicine	64	36%	36%
	Patients feel uncomfortable or do not appreciate telemedicine	49	28%	31%
**Have the cost of TB services increased or decreased at your clinic - consultation charges, testing, etc.? (N = 1996, Missing data = 15)	No difference	1,593	80%	81%
	Increased **	178	9%	6%
	Decreased	172	9%	8%
	Do not wish to respond	53	3%	5%
**Who bears the increased costs?(N = 173, Missing data = 5)	Increased prices are completely paid out of pocket by patients	134	77%	76%
	I (the provider) have borne the increased costs (e.g. by reducing profit margins)	26	15%	20*
	Support from partners (e.g. JEET)	18	10%	7%
	Other	6	3%	3%
**What are the three top reasons for costs to increase?(N = 174, Missing data = 4)	PPE	129	74%	68%
	Additional infection control	90	52%	50%
	COVID testing	83	48%	41%
	Transportation costs (personnel/products)	52	30%	25%
	Low patient load	42	24%	24%
**Are the TB test results delayed, compared to pre-COVID times?(N = 1538)	No	1,420	92%	92%
	Yes**	62	4%	4%
	Not reported	56	4%	4%
**Reasons for delayed TB test results? (N = 32, Missing data = 30)	Reduced lab capacity	13	41%	39%
	Testing priority predominantly for COVID-19 cases	13	41%	51%
	Sputum transportation	9	28%	15%
	Laboratories are closed	6	19%	28%
	Lack of proper reagents and supplies	2	6%	3%
#How are you communicating with TB patients for support and treatment adherence? (N = 1979, Missing data = 32)	Through the help of JEET	1,249	62%	38%
	In-person visits	1080	54%	44%
	I do not follow up	223	11%	28%
	Telehealth	195	10%	6%
	Community Health Workers / ASHA Workers	88	4%	7%
	Providing larger supply of TB medication for self-administration	78	4%	3%
	Video observed DOTS (VOT) or 99DOTS	35	2%	1%
	E-pharmacies & courier services	30	2%	1%
	Other	28	1%	1%
	Home visits and home delivery of medicines	17	1%	1%
#From your perspective, how has the process of care changed for your patients? (N = 1964, Missing data = 47)	No change	1,014	52%	51%
	Patient access to public facilities	708	36%	36%
	Patient transportation to clinics	282	14%	10%
	Using different clinics for TB testing	138	7%	7%
	Patient proximity to providers	120	6%	7%
	Follow-up from provider (Telemedicine)	155	8%	5%
#From your perspective, what costs have changed for the patient? (N = 1971, Missing data = 40)	No change	856	43%	42%
	Additional COVID-19 testing	688	35%	32%
	Increased transportation costs	589	30%	33%
	PPE	405	21%	29%
	Consultation fees	203	10%	7%
	Medication	147	7%	7%
	Increased price of TB testing (e.g., X-ray, diagnostic testing, etc.).)	134	7%	6%
Are there any government regulations in your town/city which are affecting your work? (N = 1987, Missing data = 24)	No	1,734	87%	87%
	Yes **	196	10%	8%
	Not reported	57	3%	4%
#What have you changed in your practice to better care for your patients as a result of COVID-19? (N = 2000, Missing data = 11)	Increased sanitation	1,787	89%	89%
	Temperature checks	1,746	87%	83%
	Spacing of appointments and social distancing	1,667	83%	82%
	PPE for staff	1,220	61%	62%
	Awareness raising in community	688	34%	29%
	Increased patient counseling and education	676	34%	25%
	Referring cases presumed positive for COVID-19	440	22%	16%
	Changes to building	345	17%	13%
	Tele-consultations text call and video	212	11%	7%
	Home consultation and delivery of medicines	39	2%	2%
	No major change	98	5%	5%
#Which of these changes can potentially improve TB diagnosis and care? (N = 1964, Missing data = 47)	Infection control measures will likely decrease TB transmission	1,565	80%	70%
	Testing for COVID-19 and TB together will likely increase TB case finding	832	42%	35%
	TB services could become integrated with other health programs	582	30%	24%
	Telemedicine use will improve access to care for TB patients	331	17%	13%
	I do not think TB care will change significantly	149	8%	13%

# Multichoice questions. Cumulative proportions of response categories for a question will exceed 100%. The proportions are calculated for each response category within a question, the denominator being the number of providers who consented (max=2011) and presented as unweighted and weighted. Weighted proportions for a particular response category might be larger than another category whose N is larger, and vice versa.

** Conditional questions based on option chosen by respondent in the preceding question. Denominator will be less than N=2011

As shown in Table 3, Age of respondent was not associated with being fully open during or after the lockdown.

**Table 3:** Predictors of providers being open during lockdown and Q1 2021.

	**Status of practice during lockdown**						**Status of practice Q1 2021**					
	**N=2392^a^**		**Odds ratio^b^**	**95% CI^b^**		**p-value**	**N=2121^a^**		**Odds ratio^b^**	**95% CI^b^**		**p-value**
	**Not open fully N=1338**	**Open N=1054**		**Lower**	**Upper**		**Not open N=430**	**Open N=1691**		**Lower**	**Upper**	
**Age**												
Less than 30 years	22	27	**Ref**				12	31	**Ref**			
30 - 45 years old	578	557	1.12	0.55	2.28	0.761	179	836	1.03	0.36	2.91	0.961
46 - 60 years old	537	381	0.96	0.45	2.01	0.905	156	656	1.26	0.45	3.52	0.657
Older than 61 years	201	89	0.65	0.29	1.44	0.288	83	168	0.64	0.22	1.9	0.425

**Provider Type**												
Health Facility	1303	960	**Ref**				418	1,576	**Ref**			
Chemist	18	71	**3.65**	**2.03**	**6.56**	**<0.001**	3	87	3.31	0.95	11.47	0.059
Laboratory	17	23	1.19	0.64	2.21	0.58	9	28	**0.36**	**0.14**	**0.95**	**0.04**

**State**												
Uttar Pradesh	418	131	**Ref**				132	328	**Ref**			
Maharashtra	178	273	**4.69**	**3.7**	**5.94**	**<0.001**	63	324	**2.03**	**1.44**	**2.87**	**<0.001**
Assam	4	20	**16.05**	**5.85**	**44.02**	**<0.001**	4	20	2.04	0.74	5.62	0.169
Gujarat	118	188	**4.82**	**3.72**	**6.24**	**<0.001**	57	193	1.32	0.93	1.87	0.12
Rajasthan	101	69	**2.11**	**1.55**	**2.87**	**<0.001**	42	115	1.05	0.71	1.56	0.79
Delhi	85	54	**2.15**	**1.56**	**2.96**	**<0.001**	30	89	1.3	0.83	2.04	0.258
Bihar	100	26	0.81	0.54	1.24	0.335	20	102	**2.02**	**1.24**	**3.29**	**0.005**
Madhya Pradesh	51	18	1.06	0.63	1.81	0.821	11	56	1.74	0.87	3.51	0.119
Tamil Nadu	54	17	0.96	0.57	1.63	0.892	22	50	0.89	0.5	1.58	0.692
Haryana	33	20	**1.91**	**1.16**	**3.15**	**0.011**	8	45	**2.24**	**1.05**	**4.78**	**0.036**
Karnataka	132	114	**1.98**	**1.44**	**2.73**	**<0.001**	22	207	**3.6**	**2.1**	**6.17**	**<0.001**
West Bengal	23	63	**5.76**	**3.27**	**10.15**	**<0.001**	11	76	2.23	0.99	5.02	0.054
Telangana	17	46	**6.08**	**3.44**	**10.76**	**<0.001**	1	56	**20.06**	**2.5**	**161.09**	**0.005**
Andhra Pradesh	15	4	0.81	0.29	2.28	0.695	5	13	1.13	0.36	3.51	0.836
Punjab	9	11	**4.01**	**1.46**	**10.97**	**0.007**	2	17	3.7	0.95	14.37	0.059

Binary variable: Open = Open for old and new patients: Not open = All other categories.

Missing responses = 20.

Binary variable: Open = Open: Not open = All other categories.

Missing responses = 24.

^a^N=Based on multi-response question. Frequencies and Odds Ratios are based on no. of responses by providers > sample size.

^b^Odds ratios and confidence intervals are weighted to account for survey design and response bias.

Logistic regression model was mutually adjusted for Age, provider and state variables.

Compared to providers in Uttar Pradesh, which had relatively moderate incidence of COVID-19 in the first wave, providers in Assam (OR: 16.1, 95 % Confidence Interval (CI): 5.85–44.02), Telangana (OR: 6.1, CI: 3.44–10.76), Gujarat (OR: 4.8, CI: 3.72–6.24), Rajasthan (OR: 2.1, 1.55–2.87), Punjab (OR: 4.0, CI: 1.46–10.97) and Haryana (OR: 1.9, CI: 1.16–3.15), all low incidence states, had higher odds of being completely open during the 2020 lockdown. However, high incidence state including West Bengal (OR: 5.8, CI: 3.27–10.15), Maharashtra (OR: 4.7, CI: 3.7–5.94), Delhi (OR: 2.2, CI: 1.56–2.96), Karnataka (OR: 2.0, 1.44–2.73), also had higher odds of being completely open during the 2020 lockdowns, compared to Uttar Pradesh. In Q1 2021, only providers in Karnataka (OR: 3.6, CI: 2.1–6.17), Maharashtra (OR: 2.0, CI: 1.44–2.87), Telangana (OR: 20.0, CI: 2.5–161.09), Bihar (OR: 2.0, CI: 1.24–3.29) and Haryana (OR: 2.24, CI: 1.05–4.78) had higher odds of being completely open compared to those in Uttar Pradesh.

Compared to health facilities, the odds of being fully open was higher for chemists (OR: 3.7, CI: 2.03–6.56) during the lockdown and lower for laboratories (OR: 0.36, CI: 0.14–0.95) in Q1 2021.

### 3.3 Restrictions in outpatient visits and TB services

**Fig. 3:**
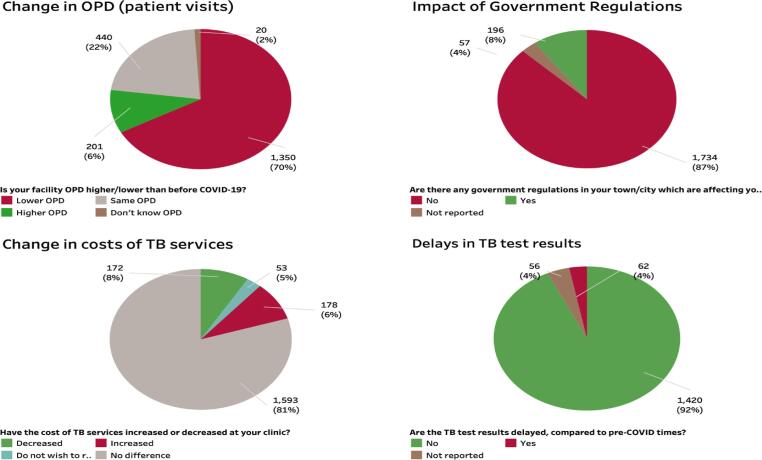
Impact on OPD load and TB services.

Majority of providers (70 %) reported that, compared to pre-COVID-19 levels, the out-patient load had decreased (Table 2 and Fig. 3a).

During the 2020 lockdown period, 40 % of surveyed providers were completely closed to all clients. However, very few providers (2 %) were closed to all clients in Q1 2021. During the lockdown, 323 (11 %) of respondents used teleconsultations (texting, phone calls, WhatsApp etc.) with their clients. This number dropped down to 122 (4 %) in Q1 2021.

There was a slight decrease (6 % reduction) in the number of providers providing TB testing (1,776 pre-COVID vs 1,661 in 2021). Less than half of TB providers (n = 960 or 33 %) were now providing or referring patients for COVID-19 testing, in compliance with the TB/COVID-19 bi-directional screening policy of the Indian government issued in August 2020 [16]. The number of providers providing TB medications dropped from 1,612 to 1,141, representing a 30 % drop.

When asked about the impact of Government regulation on their practice, 1,734 (87 %) of providers said they were no direct impact (Fig. 3b). Of the 196 (8 %) who said their practice was impacted by Government regulations, 54 (28 %) was due to the extra burden of implementing infection control and social distancing; 40 (20 %) mentioned disturbances due to constantly changing government regulations, and the additional mandatory provider registration in some states; lockdowns, facility closures and transportation challenges was mentioned by 20 (10 %); and providing on-site or referring patients for COVID-19 testing were mentioned by 19 (10 %). Four providers (2 %) were deployed out of their facilities to join the Government emergency response efforts.

Most providers (81 %, n = 1,593) reported no change in the cost of TB services (Fig. 3c). Of the 174 who gave reasons for increases in costs, 129 (68 %) attributed increases to the costs of personal protective equipment (PPE), while 90 (50 %) mentioned costs of implementing infection control measures (hand sanitation, temperature checks, appointment spacing and other measures), 83 (41 %) attributed costs to COVID-19 testing, 52 (24 %) mentioned the additional logistic burden for personnel and products, and 42 (24 %) mentioned they had to increase costs to cover for the loss from decreased patient footfalls. Majority of providers (76 %, n = 134) reported that the increase in costs were added to patient charges, while 26 (20 %) reduced their profit margins to account for these, and 18 (7 %) said the additional costs were borne by JEET.

Most providers (92 %, n = 1,420) reported that there were no delays in receiving patients’ TB test results compared to pre-COVID times (Fig. 3d).

When providers were asked if the process of care had changed, 51 % (1,014) believed there had been no changes. Among those who stated there were changes, most attributed these changes to patient access and transportation to facilities. Similarly, 856 (42 %) said there were no changes to patient costs, and those who said there were changes, attributed these to COVID-19 testing (32 %, n = 688), transportation (33 %, n = 589) and PPE (29 %, n = 405) additional costs.

### 3.4 State-wise variations in TB diagnosis, patient care costs and telehealth use

States differed in the use of TB diagnostic methods before the pandemic and in Q1 2021 (Fig. 4a), in provider perceptions of changes to process, and in costs of care for patients (Fig. 4a, Fig. 4b). Providers also differed in the use of telemedicine before the pandemic and in Q1 2021, as well as in the adaptations made in service delivery during the pandemic (Fig. 5a, Fig. 5b).

**Fig. 4a:**
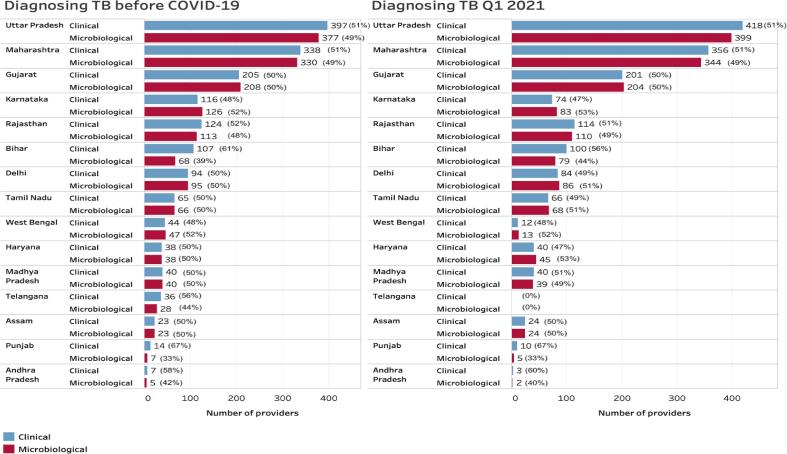
State variations in TB diagnosis – before COVID-19 and in Q1 2021.

**Fig. 4b:**
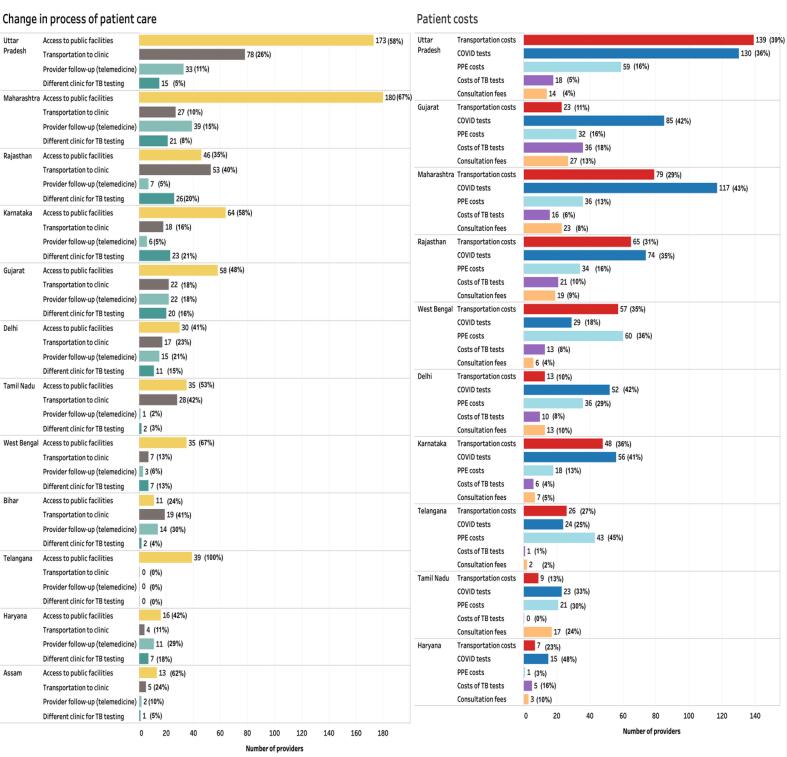
State variations in process of care and costs for patients.

**Fig. 5a:**
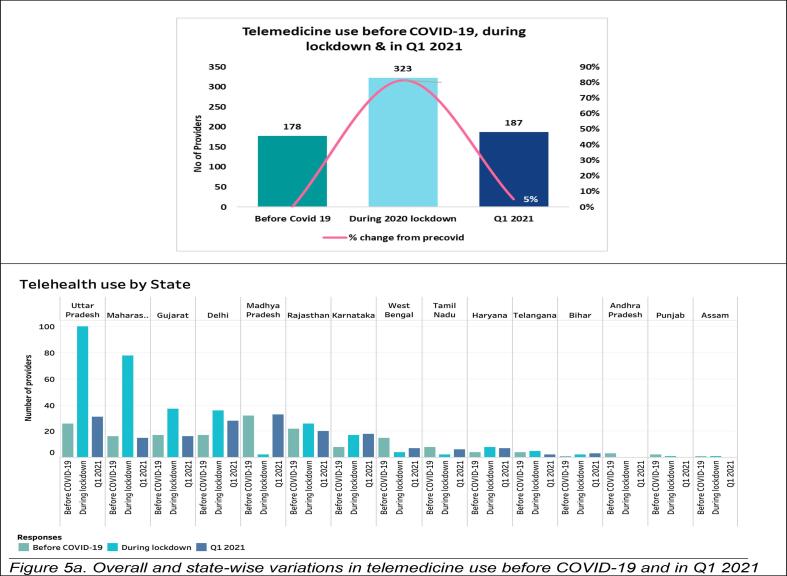
Overall and state-wise variations in telemedicine use before COVID-19 and in Q1 2021.

**Fig. 5b:**
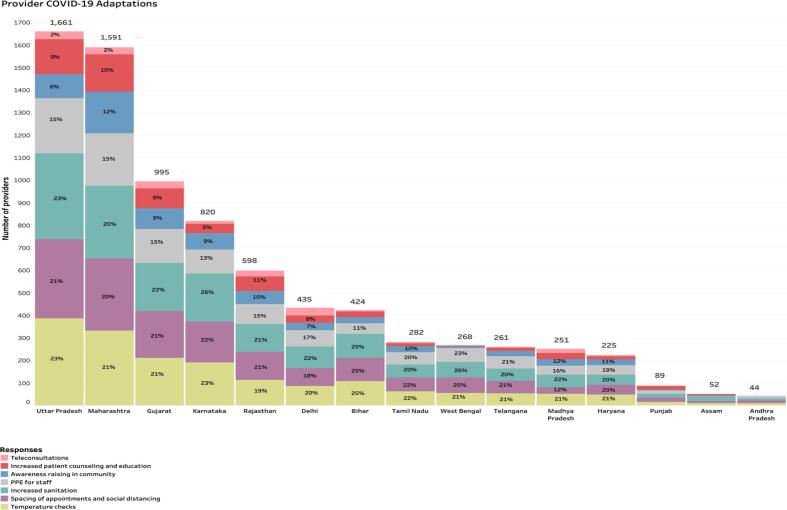
State variations in COVID-19 adaptations.

For providers who diagnosed TB before the pandemic and in Q1 2021, there were differences between and within states across the two time periods (Fig. 4a). There were no significant intra-state changes in the type of diagnostic methods used by the providers. For most of the states, providers used clinical and microbiological methods equally before the pandemic and in Q1 2021, except for West Bengal and Telangana where all diagnostic methods decreased in Q1 2021 (Fig. 4a).

Of the 950 providers (47 %) who said the process of care for patients had changed, Access to public facilities was the major change in care process in all of the states, except in Rajasthan and Bihar, where transportation to health facilities was the major change cited by providers (Fig. 4b).

In terms of patient costs, cost of transportation and COVID-19 testing costs were major issues in Uttar Pradesh, Maharashtra and Rajasthan. Cost of PPEs was a major issue in West Bengal and Uttar Pradesh. Providers cited increased cost of TB testing as the main issue in Gujarat and Rajasthan; and consultation costs in Madhya Pradesh and Gujarat (Fig. 4b).

### 3.5 Provider adaptations to COVID-19 disruptions

Compared to pre-COVID, 323 (16 %) providers said they increased use of telemedicine (teleconsultation, e-pharmacy) during the lockdown (84 % increase from 176); however, their usage dropped back to 187 in Q1 2021 (representing a 42 % drop from the lockdown period, but a 6 % increase from pre-COVID times) (Fig. 5a).

We asked the 187 providers who used telemedicine to choose the top three advantages and top three disadvantages. The main advantages of using telemedicine were ease of finding a convenient time for appointments (57 %, n = 142), elimination of transportation costs (52 %, n = 144) and ease of reaching providers by patients in remote or marginalized communities (40 %, n = 117). The main disadvantages cited were internet challenges (28 %, n = 129), absence of patient physical examination (24 %, n = 67) and lack of access to mobile phones (115 %, n = 117).

Only 98 (5 %) of providers said they had not implemented any major changes to their practice as a result of the pandemic. Among 2,000 providers who responded to the question, the major changes were in increased sanitation (89 %, n = 1,787), temperature checks (83 %, n = 1,746) and spacing of appointments to prevent patient overlaps (82 %, n = 1,667).

There were some state variations in which infection control measures were predominant (Fig. 5b). Temperature checks, appointment spacing, and increased sanitation were the main adaptations in all states, particularly in Uttar Pradesh, Maharashtra, Gujarat and Karnataka (21–26 % of providers in each state). Additionally, staff PPE use were particularly important for providers in West Bengal (23 %), Tamil Nadu (20 %) and Telangana (21 %). Providers in Maharashtra, Madhya Pradesh, Haryana and Tamil Nadu also made significant efforts at creating community awareness (10–12 % of providers in each state), compared to the other states. The proportion of providers who said they used telemedicine as a result of the pandemic were highest in Delhi (8 %, n = 35) and Madhya Pradesh (7 %, n = 17), compared to providers from other states in Q1 2021. In total numbers using telemedicine in Q1 2021, a large number of providers in Uttar Pradesh (2 %, n = 34), Maharashtra (2 %, n = 32) and Gujarat (3 %, n = 30) also said they now use telemedicine.

## 4 Discussion

Our survey of private sector health care providers after the first wave (July-December 2020), and at the beginning of the second wave in 2021, documents state-level information on the impact of the COVID-19 pandemic on private sector TB care delivery. We summarize the impact of the pandemic and restrictions on outpatient visits and TB services, the predictors of facilities remaining open during and after widespread lockdowns, state variations in TB diagnosis, patient care costs and telehealth use, and provider adaptations to the disruptions.

Our survey documents that most providers were either fully open or functioned with adjusted opening hours in Q1 2021 as the initial lockdown restrictions in their respective geographies had mostly eased by this time-period. As age was one of the earliest identified risk factors for COVID-19 disease and mortality, with the highest increase in mortality between age groups seen in patients aged 60 to 69 [17], we also looked at whether older providers respondents were more likely to remain closed during the initial lockdown and currently. However, we did not find any association between these variables during the lockdowns or afterwards.

Our regression analysis showed facility type as predictive of provider status during the lockdown periods. Relative to health facilities, chemists were more likely to have been open during the lockdown periods, hinting at specific roles and the requirements needed by different segments of the healthcare sector to provide care during the pandemic. From the beginning of the pandemic, the focus of the healthcare system was on finding and isolating COVID-19 cases. The private sector chemists, who have historically been the first source of care for most patients [6], [18], [19], [20], continued to fill this role, even as patients were reluctant or were prevented from using bigger hospitals during the lockdown [21], [22], [23]. Several sources have called for additional support for chemists, medicine vendors and other private sector providers to improve the referrals of patients into the national TB elimination program, and subsequently improving TB notifications, especially in high burden TB countries with a large share of private sector healthcare [4], [24], [25], [26]. At the start of the pandemic, private hospitals and laboratories may have required additional capacity, support, and approvals from the government to provide COVID-19 healthcare, adding to the difficulty in establishing their role during the pandemic [27], [28].

**Fig. 6:**
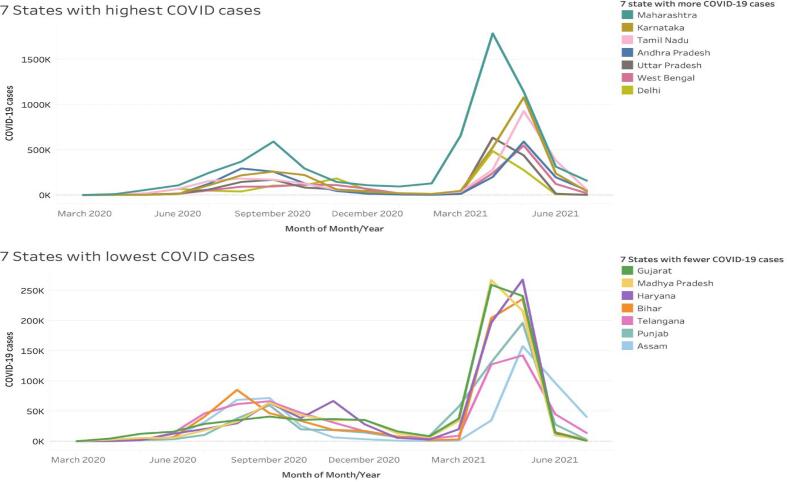
Top and Bottom COVID-19 cases.

Geographic location was also predictive of facility closures. Apart from Maharashtra, the states with highest odds of remaining open during the lockdown – Assam, Telangana, West Bengal, Gujarat and Punjab – were among the states that reported the lowest COVID-19 cases in the first wave (Fig. 6) [27]. In Q1 2021, providers in Telangana, Karnataka, Maharashtra and Bihar were more likely than other states to be fully open; and these states include both high and low COVID-19 incidence states. One reason for this could be that the JEET supported facilities in Karnataka and Telangana were in mostly urban districts, compared to the districts in our reference State Uttar Pradesh.

Similar to several other high burden TB countries, providers reported decrease in patient visits due to restrictions imposed by COVID-19 [21], [22], [23], [29], [30]. Several models had predicted up to 50 % reduction in TB care-seeking globally as a result of the pandemic [31], [32]. Data from several studies confirm that patients were avoiding hospitals due to movement restrictions, fear of contracting or being diagnosed with COVID-19 and stigma; or avoiding hospitals designated as COVID facilities. This has resulted in months of care-seeking delays particularly for infectious diseases [22], [33]. Delayed patients care-seeking for non-COVID-19 cases has increased in India as a result of the pandemic, particularly the second (Delta) wave (March-June 2021) which saw entire healthcare facilities dedicated exclusively to COVID-19 care [34], [35].

Providers in our survey indicated that the impact of the pandemic on the timeliness of TB testing was minimal. This could have been because physicians sending TB tests to private laboratories may have had fewer delays compared to public laboratories that were prioritised for COVID-19 testing, at least in the first wave. A review of data from several countries indicated decreases in TB testing and delays in diagnosis in some high burden TB settings as a result of the pandemic [22]. Data from two studies in public health facilities in Karnataka and Haryana indicate significant drops in TB specimens received for testing [36], [37], similar to our data indicating that both clinical and microbiological TB screening had decreased. Additionally, there were reports, especially during the earlier months of the pandemic, that several GeneXpert machines procured for TB testing had been reassigned to COVID-19 testing [38], [39], in line with the WHO rapid advice [40].

According to providers, costs for TB care had not changed significantly. However, where there were increases, the additional costs incurred by the patients. In low- and middle-income countries, the numbers of patients experiencing catastrophic healthcare expenditure have increased in recent years [2]. However, providers believed that the overall process of care and costs for patients had changed, mostly due to limited access to public facilities, transportation and PPE costs being the major reasons. Although the situation after the second 2nd and third waves are largely unknown, our findings agree with several studies in India and many other countries describing dramatic disruptions and increased costs for non-COVID-19 patients care, especially for immunization and infectious diseases [22], [23], [29], [33], [35], [41], [42], [43], [44].

Interestingly, most providers interviewed did not perceive any government regulation as impacting their work in Q1 2021. A few providers mentioned movement restrictions, infection control and referring patients for COVID-19 testing [45], [46]. While there were price caps put in place for COVID-19 testing in mid-2020 by several state governments in India, including Delhi, Punjab and Telangana [44], [46], [47], [48], none of these were particularly restrictive to TB consultations and testing.

Although a fraction of providers believed telemedicine use is the future of TB care (13 %, n = 331), very few (8 %, n = 187) were using any form of remote care for patients in Q1 2021. Telemedicine use was not as high as initially expected, given that that at the time the world was a year into the pandemic. There has been some evidence showing that use of telehealth improved adherence to TB medication and clinical appointments in several settings, including high burden TB countries [49], [50], [51]. An intervention in India showed that telemedicine use improved contact tracing and microbiological TB detection, and similar results were seen in other high burden countries during the pandemic [52]. One reason for this low utilization of telehealth methods could be that patients do not always have access to the devices or the connectivity needed to use them in resource-limited settings [23], [53], [54], [55]. More research might be needed to understand the barriers to the use of remote care across the cascade of TB care from the perspectives of providers.

One major finding from our survey was how well private providers have adapted to the use of PPEs, social distancing, and other forms of infection control for COVID-19. Providers also saw these infection control measures as having a place in the future of TB care. These adaptations, which have also been described elsewhere in India and other high burden TB countries [52], [56], allowed providers to continue TB services and would likely have provided an additional layer of health worker and patient protection during the subsequent waves of the COVID-19 pandemic.

Similar to TB, several other publications have detailed the deleterious impact of COVID-19 on the availability, accessibility  and affordability of healthcare provision for cancer, mental health, surgical services, diabetes and hypertension, sexual and reproductive health services, including HIV, to mention a few [57], [58], [59], [60]. Providers grappled with the diversion of resources to tackle COVID-19, reduction in revenue generation, as well modification of services based on infection control mandates [61], [62], [63].

### 4.1 Limitations

There were several limitations in our survey. First, there was a much higher proportion of providers from health facilities compared to laboratories and chemists, which may limit interpretation to facility-based findings. Second, as this survey was conducted during the pandemic, it is also possible that providers who were heavily involved in the national or state response were unable to participate. Third, since this survey was conducted as an additional task during program activities utilizing existing programmatic resources, we were unable to collect additional data on potential confounders, such as sex and socioeconomic characteristics, for all providers in our random sample. However, we adjusted our analysis for survey design and response-bias, based on available data, to present the best possible estimates. Fourth, as the survey was conducted across several states in India with variations in perceptions of participating providers and interviewers, the possibility of subjective responses, recall bias of events during the 2020 lockdown, and Hawthorne effect cannot be ruled out [64]. We attempted to mitigate these potential biases by rigorously training the JEET field staff for data collection, closely monitoring daily field activities, tracking non-response in real time and random quality checking calls to providers by the survey coordinators.

It is critical to note that our findings do not include the impact on services due to the second or third wave of COVID-19 in India in 2021. The impact of the 2021 delta variant surge in COVID-19 cases in India was catastrophic, reaching a record 403,450 new cases per day on 8 May 2021 [65]. The impact of this wave on TB services is yet to be documented, but is expected to be substantially worse than the first wave, since an estimated 3 million excess deaths may have occurred during the April to June 2021 period [66].

It also remains to be seen whether any of the positive changes observed in infection prevention practices will be sustained once COVID-19 recedes.

Despite these limitations, we believe that this study presents an important perspective on the impact of COVID-19 on private sector TB care in India. Future studies should be conducted to re-examine the impact of COVID-19 in India, particularly as a result of the second, more severe wave. As we surveyed only providers, additional research on the impact of COVID-19 from the perspectives of patients and their caregivers will complementary insight into the overall impact of COVID-19 on TB healthcare in the private sector in India.

## 5 Conclusion

Data from our survey in high-burden TB states in India indicate several challenges faced by private sector TB care providers, including limited access to facilities and transportation challenges, and implementing infection control measures. The reduction in patient footfalls and significant changes to patient process of care, have all affected TB notifications. Despite these challenges, most providers had reopened their practices in Q1 2021; most providers also reported minimal impact of government regulations and minimal increases in patient costs. In response to these challenges, providers had implemented infection control measures, and marginally increased use of remote care technologies during the lockdown. Providers believed that many of the adaptations seen have the potential for improving TB care in the future. Further studies are needed to describe the impact of the more severe second wave, the third wave, and longer-term consequences in India.

## Ethical Statement

This survey was carried out as part of the routine services within the JEET network in India. Field officers contacted providers in-person or by telephone as part of their routine service calls, and consenting providers were interviewed using a web-based survey tool.

## CRediT authorship contribution statement

**Shamim Mannan:** Conceptualization, Methodology, Writing – review & editing, Resources, Supervision. **Charity Oga-Omenka:** Methodology, Software, Validation, Formal analysis, Data curation, Writing – original draft, Writing – review & editing, Visualization, Project administration. **Akhil Soman ThekkePurakkal:** Methodology, Software, Validation, Formal analysis, Investigation, Data curation, Writing – original draft, Writing – review & editing, Visualization. **Lavanya Huria:** Software, Validation, Formal analysis, Writing – original draft, Writing – review & editing. **Aakshi Kalra:** Investigation, Resources, Writing – review & editing. **Ravdeep Gandhi:** Investigation, Resources, Writing – review & editing. **Tunisha Kapoor:** Investigation, Resources, Writing – review & editing. **Nathali Gunawardena:** Methodology, Software, Writing – review & editing. **Shekhar Raj:** Investigation, Resources, Writing – review & editing. **Manjot Kaur:** Investigation, Writing – review & editing, Supervision. **Angelina Sassi:** Validation, Writing – review & editing. **Tripti Pande:** Methodology, Writing – review & editing. **Vijayan Shibu:** Conceptualization, Methodology, Writing – review & editing, Resources, Supervision. **Sanjay Sarin:** Conceptualization, Methodology, Writing – review & editing, Resources, Supervision. **Sarabjit Singh Chadha:** Conceptualization, Methodology, Writing – review & editing, Resources, Supervision. **Petra Heitkamp:** Conceptualization, Methodology, Writing – review & editing, Resources. **Jishnu Das:** Conceptualization, Methodology, Writing – review & editing, Resources, Supervision, Funding acquisition. **Raghuram Rao:** . **Madhukar Pai:** Conceptualization, Methodology, Writing – review & editing, Resources, Supervision, Funding acquisition.

## Declaration of Competing Interest

The authors declare that they have no known competing financial interests or personal relationships that could have appeared to influence the work reported in this paper.
